# Cis- and trans-regulation by histone H4 basic patch R17/R19 in metazoan development

**DOI:** 10.1098/rsob.220066

**Published:** 2022-11-16

**Authors:** Xuedi Zhang, Xiangyu Wu, Ju Peng, Angyang Sun, Yan Guo, Pengchong Fu, Guanjun Gao

**Affiliations:** School of Life Science and Technology, ShanghaiTech University, 393 Middle Huaxia Road, Pudong, Shanghai 201210, People's Republic of China

**Keywords:** *Drosophila*, development, histone, H4 basic patch

## Abstract

The histone H4 basic patch is critical for chromatin structure and regulation of the chromatin machinery. However, the biological roles of these positively charged residues and the mechanisms by which they regulate gene expression remain unclear. In this study, we used histone mutagenesis to investigate the physiological function and downstream regulatory genes of H4 residues R17 and R19 in *Drosophila*. We found all histone mutations including R17A/E/H and R19A/E/H (R17 and R19 of H4 are substituted by A, E and H respectively) result in a range of growth defects and abnormalities in chromosomal high-order structures, whereas R17E mutation is embryonic lethal. RNA-seq demonstrates that downregulated genes in both R17A and R19A show significant overlap and are enriched in development-related pathways. In addition, Western and cytological analyses showed that the R17A mutation resulted in a significant reduction in H4K16 acetylation and male offspring, implying that the R17 may be involved in male dosage compensation mechanisms. R19 mutation on the other hand strongly affect Gpp (Dot1 homologue in flies)-mediated H3K79 methylation, possibly through histone crosstalk. Together these results provide insights into the differential impacts of positive charges of H4 basic patch R17/R19 on regulation of gene transcription during developmental processes.

## Graphical abstract

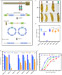

## 1. Introduction

Eukaryotic genomic DNA is packaged within the nucleus as a protein-rich complex known as chromatin. The nucleosome is the fundamental repeating unit of eukaryotic chromatin and consists of an octamer of histones with two copies of each of the histone proteins H2A, H2B, H3 and H4, around which approximately 146 base pairs of DNA are wrapped [1]. This structure not only permits compaction of the genome, but also serves as a regulatory mechanism for cellular processes such as transcription, replication and DNA repair [2–4]. Changes of chromatin, including the structure of DNA and its associated histone proteins, are key mechanisms by which transcription is regulated [5]. For example, histones are extensively decorated by covalent modifications that can affect chromatin compaction and thus influence the binding of transcription factors or recruitment of activators or inhibitors to promoters [2,6–8].

In addition to post-translational modifications (PTMs), which are major influencers of chromatin structures and functions, histones also contain basic and acidic regions, or ‘patches’, which govern nucleosome interactions and modulate their susceptibility to PTMs and their association with chromatin-binding proteins. The H4 basic patch, a positively charged patch in the H4 tail domain (residues 16-KRHR-19), contains multiple basic residues, i.e. arginine, histidine and lysine. This segment of basic residues plays an important role in regulating chromatin dynamics and multiple chromatin-modifying enzymes. For example, the H4 basic patch positively regulates the chromatin remodellers ISWI and SNF2, which affects nucleosome sliding and positioning, and thereby allows regulatory proteins access to DNA [9–11]. In yeast, the entire H4 basic patch region is required for efficient Dot1-mediated histone H3K79 methylation and plays a key role in maintaining the balance of heterochromatin domains by interacting with the silencing protein Sir3 [12,13]. Recently, the H4 basic patch was shown to regulate SAGA-mediated H2B deubiquitination and histone acetylation [14]. Furthermore, full compaction of chromatin fibres and formation of higher-order chromatin structures require amino acids 14–19 of the H4 N-terminal tail [15]. Acetylation of H4K16 regulates higher-order chromatin organization [16–19] and is required for male X-chromosome dosage compensation, male viability and ovarian germ line stem cell maintenance [20]. However, it is poorly studied whether R17H18R19 affect chromatin structures or are involved in gene regulation in animals. Considering that H18 is a weakly positively charged residue, elucidating the biological roles of the strongly positively charged R17 and R19 is more meaningful for studying the chromatin regulation of the H4 basic patch.

In this study, we demonstrate the distinct functions of positive charges in the H4 basic patch during animal development using a combination of genetic animal models, biochemical analyses and transcriptome profiling. We show that the positive charge of R17 is essential for adult survival. By inducing charge-based mutations, we identified a potential cis-regulatory link between R17 and H4K16 acetylation, as demonstrated by the H4R17A mutation, which affects H4K16 acetylation and ultimately leads to a severe sex bias. We also identified a potential trans-regulatory pathway in which R19 is required for Dot1-mediated H3K79 methylation. Our charge-based mutation strategy provides new insights into the diverse roles of different positive charges in the H4 basic patch during animal development.

## 2. Material and methods

### 2.1. Fly strains and plasmids

H4K16A, H3K79A, ubi-GFP-FRT and *His^D^* flies are available in our laboratory. Flies were grown at 25°C on standard food with 60% humidity and a 12 h light/dark cycle, or in vials placed in water baths at other indicated temperatures. Site-specific histone mutant transgenesis and fly genetics were performed as previously described [20].

The integration plasmid of attB 5xHis-GUs carrying the WT histone protein or H4R17A, H4R17H, H4R17E, H4R19A, H4R19H or H4R19E mutation was constructed using published procedures [20]. The human histone H4 cDNA sequence was used to generate wild-type constructions and H4 sequences with point mutations (R17A, R17H, R17E, R19A, R19H and R19E). The sequences were cloned into pCDH-CMV-MCS-Puro lentiviral vectors. To produce lentivirus, 293T cells were transfected with lentiviral vectors and helper plasmids (psPAX2 and pMD2.G). Supernatants containing lentivirus were collected, filtered, and concentrated at 72 h after transfection. Virus titre was determined before transduction.

### 2.2. Cell culture and generation of stable cell lines

*Drosophila* S2 cells were cultured in Schneider's *Drosophila* Medium (Gibco) containing 10% fetal bovine serum (FBS, Gibco). 293T cells were cultured in Dulbecco's modified Eagle medium (DMED, Gibco) with 10% FBS (Gibco). To generate cells stably expressing various histone mutants, cells were transduced with concentrated lentivirus (2 × 10^7^ IFU). Transduced cells were grown under puromycin selection (2 ug ml^−1^) for 48 h.

### 2.3. Antibodies

Primary antibodies against H3K4me3 (ab8580), H3K9me3 (ab8898), H3K27me3 (ab6002), H3K79me3 (Ab2621) and histone H3 (ab1791) were obtained from Abcam. Primary antibodies against histone H4 (07-108) were obtained from Millipore. Primary antibodies against H4K16ac (SC-8662-R) and LaminC (LC28.26) were obtained from Santa Cruz and DSHB, respectively.

### 2.4. Identification of lethal phases in histone mutants

Histone mutants were balanced over CyO-ActGFP to identify homozygous mutant embryos or larvae according to the absence of green fluorescent protein (GFP) expression. In total, 200 WT and 200 GFP-negative first instar larvae were collected on an apple-juice plate and tracked until adulthood. For each histone mutant, flies were counted at the second instar larval, third instar larval, pupal and adult stages. Experiments were performed in biological triplicate.

### 2.5. Developmental timing analyses

Histone mutants were balanced over CyO-ActGFP and allowed to lay eggs for 2 h on an apple-juice plate. The next day, homozygous mutant larvae lacking GFP expression were picked up in food vials. The number of animals that had pupariated was scored twice per day. Experiments were performed in biological triplicate.

### 2.6. Western blotting

*Drosophila* embryos or larvae were homogenized in phosphate-buffered saline (PBS). Protein loading buffer (Takara, 9173) was added, and the mixture was heated for 5 min at 95°C. Protein samples were loaded onto a 15% SDS-PAGE gel for electrophoresis. After transfer to a PVDF membrane and blocking in 5% dried milk dissolved in tris-buffered saline containing 0.1% Triton X-100 (TBST), protein samples were incubated with primary antisera at 4°C overnight. After three washes with TBST for 10 min, membranes were incubated with secondary antisera from rabbit or mouse at room temperature for 1 h. After three washes with TBST for 10 min, and addition of enhanced chemiluminescence substrate, western blot signals were detected with a GE AI680UV instrument.

### 2.7. DAPI staining of polytene chromosomes

Salivary glands of wandering third instar larvae were dissected in PBS, fixed in 45% acetic acid for 5 min, squashed and frozen in liquid nitrogen. Polytene spreads were stained with DAPI for DNA visualization. Micrographs were acquired using a Zeiss LSM880 inverted confocal microscope. Raw micrographs were processed with ImageJ.

### 2.8. Mosaic analysis and immunostaining

Eggs were deposited over 24 h at 25°C. Larvae were developed for 2 days and then heat shocked at 38°C for 1 h. L3 larva emerged 3 days after heat shock and were selected based on the lack of green balancer before dissection. Inverted larvae heads were fixed in PBS containing 4% paraformaldehyde (Sigma, 158 127) for 20 min at room temperature. After washing with PBS, samples were permeabilized with PBS containing 0.3% Triton X-100 for 30 min at room temperature, blocked with PBS containing 1% bovine serum albumin and 0.03% Triton X-100 for 1 h and incubated with primary antibodies diluted in PBS containing 0.1% Triton X-100 overnight at 4°C. Samples were then incubated with secondary antibodies conjugated to Alexa fluorophores (Invitrogen) before DAPI staining. Thorough washes were performed between incubations. Imaginal discs were carefully removed and mounted with VECTASHIELD Mounting Medium (VECTOR, H-1200). Micrographs were acquired using a Leica SP8 inverted confocal microscope. Raw micrographs were processed with ImageJ.

### 2.9. RNA-sequencing and data analysis

Total RNA was separately isolated from embryos collected at 6–8h and salivary glands of three instar larvae with TRIzol (Invitrogen, 15596-026). Sample quality was verified on an Agilent 2100 Bioanalyzer (Agilent). RNA-seq libraries were prepared using a NEBNext Ultra RNA Library Prep Kit for Illumina. RNA was sequenced on an Illumina NovaSeq 6000 system at Novogene. During the data-processing step, raw reads were filtered by removing adaptor sequences, reads shorter than 36bp, low-quality reads in which Phred Quality Scores of over 50% of bases is smaller than 20, and reads in which the percentage of unknown bases (N) was greater than 10%. The Q30 bases rate is about 93.5% after data pre-processing.

The cleaned data were aligned to the reference sequence using HISAT2 in default parameters. For alignment, a maximum of two mismatches were permitted. The *Drosophila melanogaster* genome and gene datasets were downloaded from FlyBase, which was used as a reference. To assess sequencing saturation, the number of genes identified was plotted against the number of cleaned reads to determine when no further genes could be detected by adding reads, which implied full saturation. To evaluate the quality of the RNA-seq dataset, the distribution of gene coverage in each sample was analysed. Gene expression levels were calculated using the Fragments Per Kilobase of transcript sequence per Million (FPKM) base pairs sequencing method. R package DESeq2 [21] was used to identify differentially expressed genes between two samples with biological replicates. In this approach, the *p*-value corresponds to the differential gene expression test, and the FDR is a method to determine the *p*-value threshold for multiple tests. |log2(FoldChange)| ≥ 1 and FDR ≤ 0.05 were used as the threshold of significance for differences in gene expression.

### 2.10. RT-qPCR

Total RNA was extracted with TRIzol following the manufacturer's instructions and treated with DNase. In total, 1 mg RNA was reverse-transcribed using PrimeScript RT Master Mix (Takara, RR036A). Quantitative PCR analyses were performed with a QuantStudio 7 detection system (Life Technologies). Primer sequences are provided in Supplemental electronic supplementary material, table S1. To quantitate the expression levels, CT values of the endogenous reference gene rp49 were included. Each experiment was performed with biological triplicates and technical duplicates.

### 2.11. GST-H4 binding assays

BL21 cells expressing GST (pGEX-4T-1) or GST-H4 tails (WT, H4R17A, H4R17E, H4R19A and H4R19E) were grown to mid-log phase, induced by treatment with 0.4 mM IPTG for 4 h at 25°C, and harvested. Cell pellets were lysed by sonication at 4°C in 200 µl lysis buffer (50 mM Tris-Cl at pH 8.0, 300 mM NaCl, 1 mM PMSF and 1 µg ml^−1^ each of leupeptin, aprotinin and pepstatin). Cell lysates were clarified by centrifugation at 14 000 rpm for 5 min at 4°C. The supernatants contained soluble protein. To isolate GST and GST-H4 tails, 10 µl of a 50% slurry of glutathione agarose (Sigma, G4510) in PBS was added to the soluble fraction and incubated for 1 h at 4°C. Thereafter, GST- and GST-H4 tail-bound agarose was pelleted, washed three times with lysis buffer for 5 min, and resuspended in 15 µl lysis buffer. Of this final slurry, 1.5 µl was analysed by SDS-PAGE to normalize GST and GST-H4 protein levels for binding reactions. *Drosophila* S2 cells were transfected with a plasmid bearing the Gpp-flag fusion, incubated for 3 days at 25°C and lysed by sonication at 4°C in 200 µl lysis buffer.

Binding reactions were performed by incubating 4 µl GST-H4 tail-bound glutathione agarose beads with 20 µl Gpp-flag lysates in a final reaction volume of 200 µl. The final buffer contained 50 mM Tris-Cl (pH 8.0), 150 mM NaCl, 1 mM PMSF and 1 µg ml^−1^ each of leupeptin, aprotinin and pepstatin. Ten microlitres of each reaction was removed as a control for Gpp input. Binding reactions were incubated with rotation for 2 h at 4°C. Beads were washed with a modified RIPA buffer (50 mM Tris at pH 7.4, 75 mM NaCl, 1% NP-40, 0.5% deoxycholate, 0.1% SDS, 1 mM PMSF and 1 µg ml^−1^ each of leupeptin, aprotinin, pepstatin) three times for 5 min. After washing, beads were resuspended in 12 µl of 4× SDS-PAGE sample buffer. Samples were loaded onto a 12% SDS-PAGE gel and probed for Gpp-flag using an anti-flag antibody (ABclonal, AE005).

## 3. Results

### 3.1. The positive charge of H4R17 is essential for development

**Figure 1.**
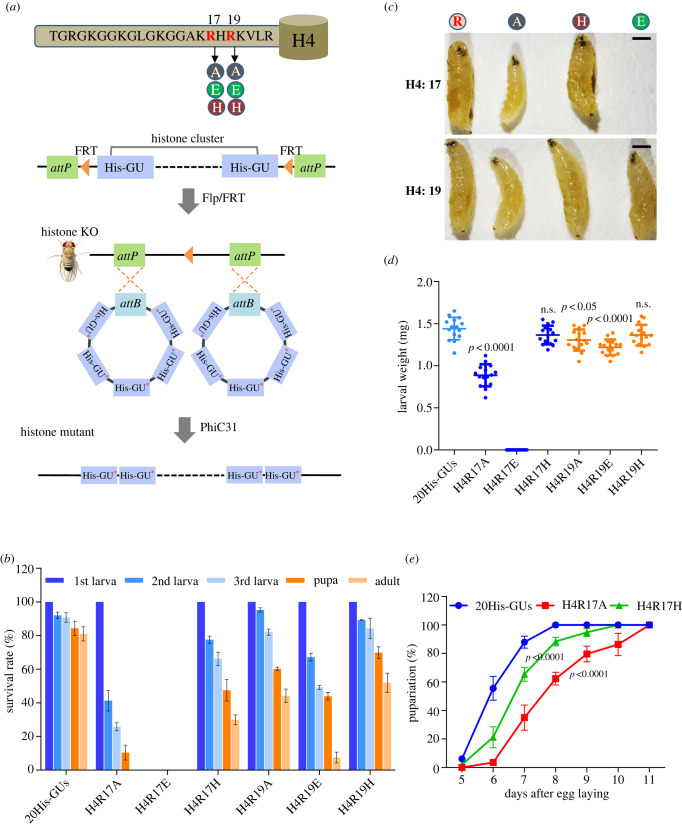
The positive charge of histone H4R17 is essential for development. (*a*) Schematic of histone mutant *Drosophila* constructs. Genomic double integrated histone mutants were constructed using the phiC31 mediated attB / attP recombination system via two attP sites on one chromosome of *His^D^* fly. The H4R17 and H4R19 mutation sites are shown. (*b*) 200 GFP-negative first-instar larvae were selected for survival tracing on the apple-juice plates until adulthood stage. For each histone mutant, the number was counted for second-instar larvae, third-instar larvae, pupae and adult stages. (*c*) WT, H4R17A, H4R17H, H4R17E, H4R19A, H4R19H and H4R19E mutant larvae at 5.5 days after egg laying (AEL). Scale bar = 1 mm. (*d*) Weight in milligrams of H4R17 and H4R19 mutant larvae at 5.5 days. Each point represents a larva. The horizontal bar indicates the average weight of the given genotype larvae. Error bar represents SEM. ns stands for not significant. (*e*) Time taken for animals to pupariate for WT (*N* = 182), H4R17A (*N* = 185) and H4R17H (*N* = 105). All data acquired from at least three independent experiments and represented as mean ± SEM.

Histone gene units (HisGU) reside in a tandem array in the *Drosophila* genome, and transgenic reintroduction of 20 copies of wild-type (WT) HisGU is sufficient to revert histone deficiency (HisD) to a WT phenotype [20]. To demonstrate the roles of the H4 basic patch in *Drosophila*, we used the previously described histone mutagenesis platform to induce charge-based mutations of R17 and R19 of H4 (R17 and R19 are substituted by neutrally charged alanine (A), positively charged histidine (H), and negatively charged glutamate (E), respectively), and obtained H4R17A, H4R17H, H4R17E, H4R19A, H4R19H and H4R19E mutants (figure 1*a*; electronic supplementary material, figure S1A, B) [20]. Each mutant had 20 identical amino acid substitutions in the diploid genome (electronic supplementary material, figure S1C). Molecular validation confirmed the genotypes of the mutant flies (electronic supplementary material, figure S1D). In adult progeny of experimental crosses obtained at 25°C, we did not obtain flies carrying the H4R17A or H4R17E mutation. However, approximately 30% of the first instar larvae of H4R17H survived to adulthood (figure 1*b*), indicating that the positive charge of R17 is essential for *Drosophila* development. Further examination revealed that a negatively charged substitution of R17 (H4R17E) resulted in embryonic lethality (figure 1*b*). Interestingly, the offspring of heterozygous parents comprised about 42% H4R19A, 51% H4R19H, and 9% H4R19E homozygous flies (figure 1*b*), suggesting that a positive charge at position 19 of H4 is, to some extent, dispensable for survival of *Drosophila*.

To further explore the biological function of R17/R19 in the H4 basic patch at the developmental level, we examined the weight and morphologies of third stage larvae at the same developmental period and quantified the developmental timing of H4R17 and H4R19 homozygous mutants. Comparison of age-matched larvae at 5.5 days after egg laying revealed that H4R17A mutants weighed less and had markedly smaller bodies than control larvae (20His-GUs) (figure 1*c,d*). Additionally, other mutants (H4R17H, H4R19A, H4R19H, and H4R19E) showed slight weight loss and smaller body sizes than control larvae. It took an average of 6, 7 and 8 days for 50% of control (20His-GUs), H4R17H homozygous mutant, and H4R17A homozygous mutant larvae to pupariate, respectively (figure 1*e*). These data indicate that the positive charge of R17 is more important than the positive charge of R19 for growth and development.

### 3.2. The positive charge of R17/R19 is essential for maintaining chromatin structure

**Figure 2.**
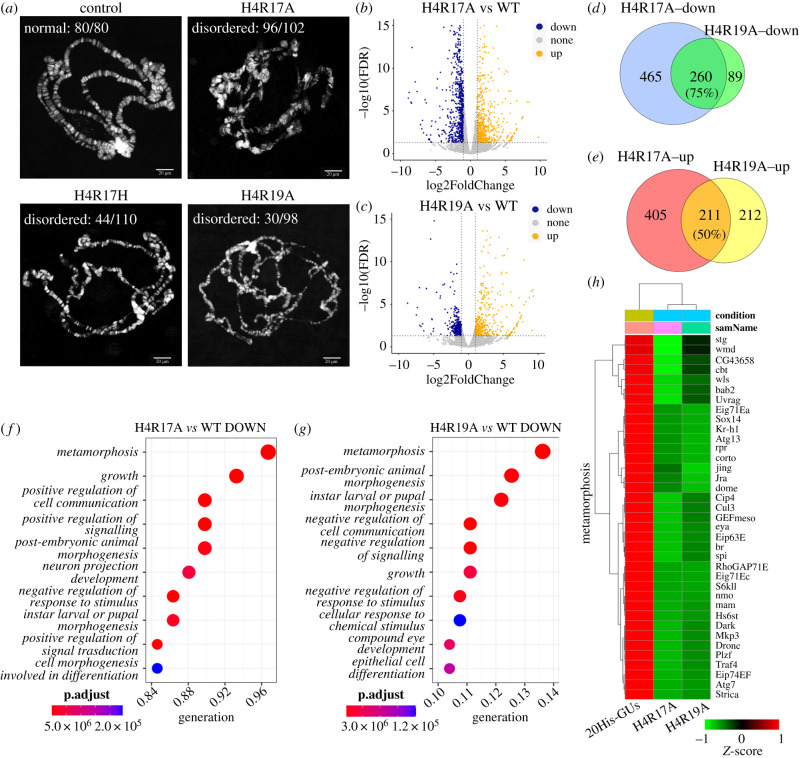
Positive charge of H4 R17/R19 is essential for maintaining chromatin structure and development associated gene expression. (*a*) Salivary glands from control (20His-GUs), H4R17A, H4R17H and H4R19A L3 larvae were squashed, and polytene spreads were stained with DAPI. The rate in the figures calculated as follow: the number of normal or disordered salivary gland samples / the number of total salivary gland samples observed. Scale bar = 20 *μ*m. (*b*) Transcriptome comparisons of salivary glands between H4R17A mutants and 20His-GUs. |log2(FoldChange)| ≥ 1 and FDR ≤ 0.05 were used as the threshold of significance for differences in gene expression. (*c*) Transcriptome comparisons of salivary glands between H4R19A mutants and 20His-GUs. |log2(FoldChange)| ≥ 1 and FDR ≤ 0.05 were used as the threshold of significance for differences in gene expression. (*d,e*) Venn diagram showing overlap between the differentially expressed genes in H4R17A and H4R19A in (*b*) and (*c*). (*f*) Gene ontology categories enriched using genes significantly downregulated in H4R17A. (*g*) Gene ontology categories enriched using genes significantly downregulated in H4R19A. (*h*) Heatmap of differentially expressed genes that belong to metamorphosis GO term in panel *f* and *g*. Red denotes high expression values, and green denotes low expression values. Heatmap of the genes expression level (FPKM) using Z-score (ranged from −1 to 1) for normalized value.

According to the X-ray structure of nucleosomes [22], direct alterations of positive charges of histones can directly affect chromatin structures and thus influence gene transcription. To test whether loss of the positive charges of R17 and R19 affects chromatin structures, we cytologically analysed the polytene chromosome structure in salivary glands from H4R17A, H4R17H, H4R19A and WT flies. There were no detectable abnormalities in WT larvae (figure 2*a*). Notably, >90% of salivary glands (96/102) from third instar larvae with the H4R17A mutation contained structurally disorganized polytene chromosomes (figure 2*a*). Moreover, 40% (44/110) of H4R17H mutants had abnormal chromosome structures (figure 2*a*). In addition, we observed polytene chromosomal abnormalities in ∼30% of H4R19A mutants. These results suggest that positive charges of the H4 basic patch, especially R17, are necessary for the maintenance of higher-order chromatin structures.

### 3.3. The positive charge of R17/R19 is essential for development-associated gene expression

To investigate the impact of genome-wide expression caused by disruption of the chromatin structure in H4R17A and H4R19A mutants, we performed RNA-sequencing (RNA-seq) of salivary glands obtained from H4R17A, H4R19A and WT (20His-GUs) third instar larvae. Compared with WT larvae, approximately 1341 genes (5% false discovery rate [FDR]) were dysregulated in the H4R17A mutant, of which 725 were downregulated and 616 were upregulated (figure 2*b*). We identified 772 dysregulated genes (5% FDR) in the H4R19A mutant, of which 349 were downregulated and 423 were upregulated (figure 2*c*). These results demonstrate that a positive charge at R17 has more pronounced effects on genome-wide transcription than a positive charge at R19, which may explain why the H4R17A mutation induced a more severe growth defect than the H4R19A mutation (figure 1*c*).

To investigate whether the positive charges of R17 and R19 are redundant for regulating gene expression, we first compared differentially expressed genes between R17 and R19 mutants. We found that 75% of downregulated genes (260/349) in the H4R19A mutant were also downregulated in the H4R17A mutant (figure 2*d*), whereas 50% of upregulated genes in the H4R19A mutant were also upregulated in the H4R17A mutant (figure 2*e*). These data suggest that R17 and R19 regulate downstream genes via similar mechanisms. To define the pathways affected by the H4R17A and H4R19A mutations, we performed gene ontology (GO) analyses of genes dysregulated in the H4R17A and H4R19A mutants using the clusterProfiler package (figure 2*f,g*) [23]. Downregulated genes in the H4R17A mutant were mainly enriched in developmentally relevant pathways, including metamorphosis, growth and morphogenesis (figure 2*f*). We assessed 485 genes with GO terms including ‘metamorphosis’. As indicated by the heat map of hierarchical clustering analysis, 56 of the 485 metamorphosis-related genes were differentially expressed in the H4R17A mutant compared with the WT. These differentially expressed genes included Eig71Ec, Eig71Ea, Eip63E, Eip74EF, br and Sox14, which mediate puparium formation [24–28] (figure 2*h*), and Hr4, dco, mnb, Pdk1, shot and pico, which mediate *Drosophila* growth (electronic supplementary material, figure S2A) [29–34]. Downregulated genes in the H4R19A mutant were also enriched for developmentally related pathways similar to the H4R17A mutant (figure 2*g*). Interestingly, as shown in the heatmap of hierarchical clustering analysis of the ‘metamorphosis’ pathway, the H4R17A mutation had a greater effect on downregulated gene expression than the H4R19A mutation (figure 2*h*). This may explain why the H4R17A mutation caused more severe developmental delay defects. Moreover, upregulated genes induced by R17 and R19 mutations were enriched in many metabolic pathways (electronic supplementary material, figure S2B–D). Overall, these findings suggest that positive charges of the histone H4 basic patch play an important role in animal development and growth.

### 3.4. R17, not R19, is required for male X-linked dosage compensation

**Figure 3.**
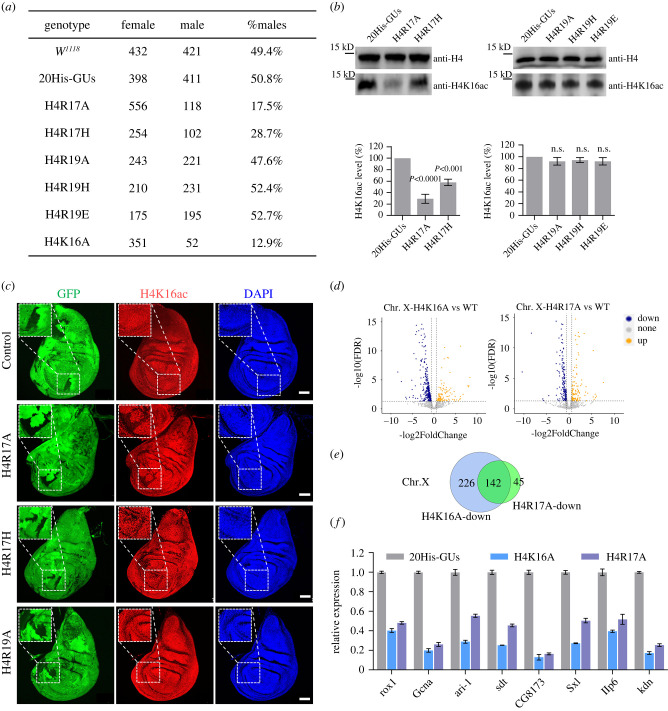
R17, not R19, in H4 basic patch, is required for male X-linked dosage compensation. (*a*) The number of male and female progeny larvae from control and histone mutant flies. (*b*) Western blots of whole-cell extracts using specific antibodies show the status of H4K16ac in larvae expressing wild-type histones or larvae expressing the indicated histone mutation. Antibodies directed against histone H4 serve as loading control. Densitometry of western blot bands was quantified with Image J software. Values are means ± SEM of three biological replicates. n.s. stands for not significant. (*c*) Wing imaginal discs of the indicated histone replacement genotype. Immunostaining with the GFP (green) and H4K16ac (red) antibodies is shown. DNA was stained with DAPI (blue). Clones of interest were marked by the lack of GFP signal and are indicated by dashed lines. Scale bar = 50 *μ*m. (*d*) Transcriptome comparisons of salivary glands X chromosome genes between H4K16A, H4R17A mutants and 20His-GUs. |log2(FoldChange)| ≥ 1 and FDR ≤ 0.05 were used as the threshold of significance for differences in gene expression. (*e*) Venn diagram showing overlap between the differentially downregulated genes in H4R17A and H4K16A in (*d*). (*f*) RT-qPCR verification of downregulated genes in H4K16A and H4R17A mutants. Eight X-linked genes (rox1, Gcna, ari-1, sdt, CG8173, Sxl, Ilp6 and kdn) were chosen as the targets. Values are means ± SEM of three biological replicates (rp49 was the reference gene for normalization; RNA was extracted from salivary gland).

Transcriptome profiling revealed a partial co-regulatory link between R17 and R19 in the developmental pathway; therefore, we hypothesized that mutations that change the charges of these residues might affect the biological function of H4K16 due to neighbour steric hindrance effects [35]. H4K16 acetylation mediated by MOF–MSL complexes contributes to 2-fold transcriptional activation for X-chromosome dosage compensation [36]. To investigate whether the R17-R19 basic patch is involved in activation of X-chromosome genes in males, we first examined the male: female ratio of homozygous mutants. The H4R17A mutation was lethal in adults; therefore, we calculated the male: female ratio of third instar larvae. Interestingly, among randomly tested H4R17A mutant larvae, 17.5% (118/674) were male (figure 3*a*), yielding a male: female ratio of 1:5. Similarly, 12.9% of H4K16A mutant larvae were male (figure 3*a*). However, the male: female ratio of R19 mutants (H4R19A, H4R19H and H4R19E) was approximately 1:1 (figure 3*a*). The male lethality observed with the H4R17A mutation is consistent with the 80% male adult lethal phenotype that we previously described with the H4K16A mutation [20].

To further investigate how mutations that change the charges of the basic patch affect male viability, we examined H4K16ac levels in third instar larvae with R17 (H4R17A and H4R17H) and R19 (H4R19A, H4R19H and H4R19E) mutations by western blot analysis. The H4K16ac level was approximately 75% lower in the H4R17A mutant than in the control (20His-GUs), whereas the compensatory substitution mutation to positively charged H (H4R17H) restored the H4K16ac level to 60% of that in the WT (figure 3*b*). However, the three charge mutations of R19 (H4R19A, H4R19H and H4R19E) did not significantly decrease the H4K16ac level (figure 3*b*). This finding suggests that maintenance of normal H4K16ac levels is dependent on the positive charge of R17 but not R19. This may help to explain why the positive charge of R17, but not R19, is important for male survival.

To investigate crosstalk between R17 and H4K16ac, mosaic analyses of histone mutants were performed using FLP-FRT-mediated recombination [20] (electronic supplementary material, figure S3A). Homozygous HisD clones were detected when they are supplemented with 20*Χ* HisGUs of WT or mutated histones in larval tissues [20]. We adopted this system to examine H4K16ac levels in WT, H4R17A, H4R17H and H4R19A flies. Consistent with the western blot results, the H4K16ac level was markedly decreased across the wing disc of H4R17A mutants but was weakly decreased in H4R17H mutants (figure 3*c*). Staining of H4K16ac in the wing disc of H4R19A and WT flies was indistinguishable from that in neighbouring tissues (figure 3*c*).

To gain further insights into whether the H4R17A mutation affects male X-chromosome dosage compensation, we compared differentially expressed genes on the X-chromosome between H4R17A and H4K16A mutants. In H4K16A male flies, 16.5% (368) of X-linked genes were significantly downregulated compared with WT controls (figure 3*d*). Transcriptome analysis revealed that the H4R17A mutation resulted in downregulation of 187 X-linked genes, 76% (142) of which overlapped with genes downregulated in the H4K16A mutant (figure 3*e*). We confirmed the downregulation of eight known X-linked genes in H4K16A and H4R17A male flies relative to WT controls by RT-qPCR [37–44] (figure 3*f*). For example, expression of *rox1* (long non-coding RNA on the X-chromosome), which is required for male dosage compensation [45], was greater than 50% lower in H4R17A and H4K16A mutants than in the WT. To understand whether these downregulated X-linked genes presented in both H4K16A and H4R17A mutants are the direct targets of H4K16ac, we analysed the published anti-H4K16ac ChIP-seq data from salivary glands of male third instar larvae [36]. We found that 83% (307/368) of downregulated X-linked genes in H4K16A had the H4K16ac ChIP peaks and were therefore likely to be direct targeted genes regulated by H4K16ac (electronic supplementary material, figure S3B). 65% (121/158) of downregulated X-linked genes in H4R17A were enriched with H4K16ac peaks, while overlapping with downregulated genes in H4K16A (electronic supplementary material, figure S3C). Thus, the downregulation of these X-linked genes in H4R17A mutant is most likely due to the reduced modification of H4K16ac.

Furthermore, global transcriptome analysis demonstrated that 65% of genes upregulated in the H4R17A mutant were also upregulated in the H4K16A mutant (electronic supplementary material, figure S3D, E), while 70% of genes downregulated in the H4R17A mutant were also downregulated in the H4K16A mutant (electronic supplementary material, figure S3F). GO analyses suggested that dysregulated genes in the H4R17A and H4K16A mutants were enriched in very similar terms (figure 2*f*; electronic supplementary material, figure S2B, S3G, and S3H). For example, H4K16 and R17 co-regulated expression of genes involved in metamorphosis and ribonucleoprotein complex biogenesis (figure 2*f*; electronic supplementary material, figure S2B, S3G and S3H). All these findings suggest that potent cis-regulatory crosstalk exists between H4K16 and R17.

### 3.5. R19 trans-regulates H3K79 methylation

**Figure 4.**
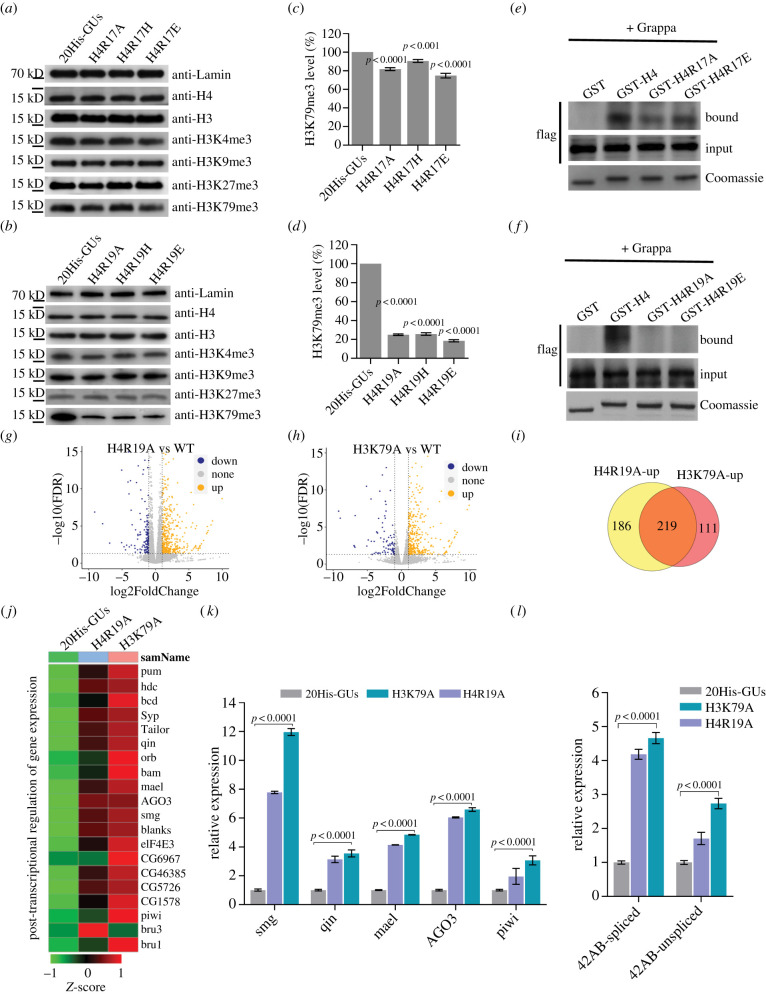
Histone H4 arginine 19 trans-regulates H3K79 methylation. (*a,b*) Western blots of whole-cell extracts using specific antibodies show the status of H3K4me3, H3K9me3, H3K27me3, H3K79me3 and H4K16ac in embryos expressing wild-type histones or indicated mutation. Antibodies directed against histone H3, H4 and LaminC serve as loading controls. (*c,d*) Densitometry of western blot bands of H3K79me3 shown in panel *a* and *b* was quantified with Image J software. Values are means ± SEM of three biological replicates. (*e,f*) GST-H4 peptide fusion pull-downs were performed as described previously. Recombinant-purified grappa-flag was incubated in the presence of a GST control, or a GST-H4 tail peptide fusion encoding residues 1–34 of histone H4 (GST-H4_1–34_) or GST-H4_1–34_ with indicated mutation. Bound grappa-flag was detected by flag antibodies (Bound). Reaction inputs were probed with flag antibodies to confirm equivalent amounts of grappa protein (Input). GST-histone constructs were Coomassie-stained to indicate the amount of GST histone fusion protein loaded per lane. (*g*) Transcriptome comparisons of embryos between H4R19A mutants and 20His-GUs. |log2(FoldChange)| ≥ 1 and FDR ≤ 0.05 were used as the threshold of significance for differences in gene expression. (*h*) Transcriptome comparisons of embryos between H3K79A mutants and 20His-GUs. |log2(FoldChange)| ≥ 1 and FDR ≤ 0.05 were used as the threshold of significance for differences in gene expression. (*i*) Venn diagram showing overlap between the differentially upregulated genes in H4R19A and H3K79A in (*g*) and (*h*). (*j*) Heatmap of differentially expressed genes from H4R19A and H3K79A that belong to the GO terms (post-transcriptional regulation of gene expression). Red denotes high expression values, and green denotes low expression values. Heatmap of the genes expression level (FPKM) using Z-score (ranged from −1 to 1) for normalized value. (*k,l*) RT-qPCR verification of genes expression in H4R19A and H3K79A mutants. smg, qin, mael, AGO3 and piwi were chosen as the targets in panel *k*. 42AB piRNA cluster was chosen as the targets in panel *l*. Values are means ± SEM of three biological replicates (rp49 was the reference gene for normalization; RNA was extracted from embryos).

Charge-based mutations of the basic patch broadly affect global gene expression according to transcriptome profiling. Therefore, we next investigated whether there is putative crosstalk between the H4 basic patch and other epigenetic signatures of gene silencing or activation. We examined the expression levels of several known important histone modifications (H3K4me3, H3K9me3, H3K27me3 and H3K79me3) in R17 and R19 mutants at the embryonic developmental stage (figure 4*a,b*). Western blot analysis of whole embryo extracts showed that when R17 was mutated to A, H or E, the levels of H3K4me3, H3K9me3 and H3K27me3 were unaffected compared with the WT (figure 4*a*). However, the level of H3K79me3 was slightly decreased in the H4R17A and H4R17E mutants (figure 4*c*). The levels of H3K4me3, H3K9me3 and H3K27me3 were not markedly decreased in the H4R19A and H4R19E mutants (figure 4*b*). However, mutation of R19 to A, H or E reduced the level of H3K79me3 by approximately 80% compared with the WT (figure 4*b,d*), indicating putative crosstalk between R19 and H3K79. To determine whether this crosstalk is conserved in other tissues, we examined H3K79me3 levels in the salivary glands of R19 mutants. The level of H3K79me3 was markedly lower in H4R19A and H4R19E mutants than in the WT, similar to the results obtained in embryos (electronic supplementary material, figure S4A).

The interaction between Dot1 and the H4 N-terminal tail is essential for H3K79 methylation [46]. Therefore, we further examined the requirement of R19 for Gpp (Dot1 homologue in flies)-mediated generation of H3K79me3. We performed *in vitro* binding assays in which GST-H4_1–34_ was incubated with recombinant WT Gpp. Western blotting detected binding of Gpp to GST-H4_1–34_ (figure 4*e,f*). By contrast to the slight decrease in the interaction between Gpp and H4_1–34_ when R17 was mutated to A or E (figure 4*e*), mutation of R19 to A or E almost totally abolished binding of Gpp to the H4 N-terminal tail (figure 4*f*). All these data indicate that the interaction between Gpp and the H4 basic patch strongly depends on the arginine at position 19 of H4.

We further explored which genes are trans-regulated by crosstalk between R19 and H3K79 during early embryonic development. RNA-seq of H4R19A mutant embryos identified 567 dysregulated genes (5% FDR), of which 162 were downregulated and 405 were upregulated (figure 4*g*). In H3K79A mutants, we identified 416 dysregulated genes (5% FDR). Among these, 43% of downregulated genes (37/86) overlapped with downregulated genes in H4R19A mutants, while 66% of upregulated genes (219/330) overlapped with upregulated genes in H4R19A mutants (figure 4*h,i*; electronic supplementary material, figure S4B). GO analysis revealed that many co-upregulated genes were enriched in the ‘post-transcriptional regulation of gene expression’ cluster based on a comparison of both datasets (electronic supplementary material, figure S4C, D), including Smg, Qin, Ago3, Mael and Piwi [47–51] (figure 4*j*), which are involved in the piRNA pathway. To further delineate the possible role of trans-action between R19 and H3K79 in the piRNA pathway, we validated the expression of these co-regulated substrate genes and piRNAs by RT-qPCR. Consistently, these co-regulated genes and piRNAs were markedly upregulated in H3K79A and H4R19A mutants (figure 4*k,l*), suggesting that trans-histone crosstalk between R19 and H3K79 may play an important role in silencing of the PIWI-piRNA pathway.

## 4. Discussion

Although structural and biochemical studies suggest that the histone H4 basic patch regulates the binding and activity of chromatin-binding factors on nucleosome surfaces, few studies have directly elucidated its roles in biological functions and transcriptional regulation in metazoans. In this report, we utilized a previously published method to construct *Drosophila* mutant models bearing amino acid charge alterations in the histone H4 basic patch. We further elucidated the functional differences of the positively charged residues of this basic patch in animal development and their involvement in the regulation of gene transcription through cis and trans-mechanisms. Based on the growth and developmental phenotypic defects observed upon substitutions of R17 and R19 in the histone H4 basic patch with A, E or H, we concluded that the positive charge at position 17 plays a more critical role than the positive charge at position 19 during development. Interestingly, we identified cis-acting regulation between R17 and H4K16 acetylation as well as trans-acting regulation between R19 and H3K79 methylation. Furthermore, in human cultured cell lines, we investigated the finding that overexpression of H4R17A mutated proteins remarkably reduces H4K16 acetylation levels and that overexpression of H4R19A also leads to a reduction in H3K79 methylation levels (electronic supplementary material, figure S5A, B). These results suggest that cis - and trans regulation between R17 and H4K16 acetylation and between R19 and H3K79 methylation in *Drosophila* may be conserved in other eukaryotic species. This strongly indicates that positive charges of the histone H4 basic patch affect downstream processes (e.g. transcription) through complex histone crosstalk, and contribute to regulation of chromatin structures during development.

Hierarchical packaging of eukaryotic chromatin plays a central role in transcriptional regulation and other DNA-related biological processes [52]. Although the cryo-electron microscopy structure of the nucleosome *in vitro* suggests that single mutations in the basic patch, such as H4K16A, H4R17A and H4R19A, do not affect 30 nm chromatin fibre folding [53], cytological examination of polytene chromosomes revealed disorganized structures in H4R17A and H4R19A mutant flies, particularly in the former. This may be due to several reasons. First, the H4 tail of nucleosomes interacts with acidic patch regions on the surface of adjacent nucleosomes to support compaction of the chromatin fibre [1,15,54]. When the positive charge of the H4 basic patch is altered, the internucleosomal interaction is weakened, possibly leading to chromatin decompaction. Second, the basic patch is required for DNA accessibility by chromatin-binding proteins such as ISWI, Snf2 and Chd1 [9–11,55–57]. Loss of positive charges in the H4 basic patch likely affects chromatin remodelling. Thus, we propose that positive charges in the H4 basic patch are essential for regulating higher-order chromatin structures, which is key for transcriptional regulation. Downregulated genes upon alteration of positive charges in the basic patch were ontologically enriched mainly in growth-, metamorphosis- and morphogenesis-related pathways, which further explains the developmental defects of H4R17A and H4R19 mutants. However, it is unclear why alterations of positive charges specifically affect genes involved in growth and development, and this merits future exploration.

Another question is whether important epigenetic modifications of R17 and R19 occur *in vivo*. Although *in vitro* biochemical experiments showed that R17 and R19 can be methylated [58], the presence of such modifications has not been examined *in vivo*. In this study, substitution of R17 with a positively charged H amino acid partially rescued the lethal phenotype observed with the A and E point mutations, suggesting that the positive charge itself, rather than modifications, may be essential for survival of multicellular organisms. However, these results do not exclude the possibility that R17 and R19 are modified *in vivo*, and the biological significance of such modifications requires further investigation.

### 4.1. Cis-crosstalk between R17 and H4K16

R17 carries a positive charge adjacent to H4K16 and can predictably influence H4K16ac based on charge steric hindrance. Although H4R17A mutation causes approximately 75% lower of H4K16ac level, RNA-seq data showed no significant differences in the expression of male-dosage related genes such as MOF, MSL1, MSL2, MSL3, and MLE between H4R17A and WT flies. This suggests that the point mutation of H4R17 did not result in decreased expression of components of the MOF-MSL complex. We reasoned that mutation of R17 might affect the binding or enzymatic activity of MOF, a histone acetyltransferase that specifically targets H4K16 [59]. MOF is abundantly localized on the X-chromosome in male *Drosophila* [60]; therefore, we examined its localization in H4R17A male *Drosophila* chromosomes. MOF protein still localized to the X-chromosome (data not shown), suggesting that charge-based mutation of R17 does not significantly affect binding of MOF to the X-chromosome. The positive charge of R17 may be required for the full catalytic function of MOF to acetylate H4K16 and form the activated conformation. In addition, the H4R17A mutation may induce the binding or activity of the H4K16ac deacetylase. Regardless, our results show that mutations of R17, but not R19, significantly decrease the H4K16ac level and downregulate many male X-linked genes (including the X dosage compensation gene *rox1*), which may explain why the male: female ratio was dysregulated in H4R17A mutants. However, we do not know whether downregulation of these X-linked genes in H4R17A male mutants is directly due to loss of the positive charge of R17, failure of H4R17A to facilitate MOF-mediated H4K16ac, or both. We propose that R17 plays an essential role in male X-chromosome dosage compensation, probably by acting synergistically with H4K16ac in cis.

### 4.2. Trans-crosstalk between the H4 basic patch and H3K79

In this study, R17 and R19 mutations variably affected H3K79 methylation by interfering with binding of the H3K79 methyltransferase Gpp [46] to the H4 N-terminal tail. Obviously, our results showed that R19 is more important than R17 for H3K79me3 during *Drosophila* embryonic development, probably because R19 residue plays a critical role in stabilizing the active-state of H3K79 catalyzed by Dot1 by forming direct interactions with the backbone atoms of H3 residues K79, T80 and Q76 [61]. Whereas residue R17 is oriented in a position to project into a deep acidic pocket of Dot1[61], such that the charge change of R17 may partially affect the H4 N-tail binding of Dot1. It is also worth mentioning that previous studies showed that H4K16ac and H4R17 are important for Dot1-mediated H3K79 methylation in yeast [12,62]. While in *Drosophila*, the H4R17A mutation resulted in a dramatic decrease in H4K16ac but less on H3K79me3. This is probably because the mode of Gpp binding to the K16 position in the H4 N-tail in *Drosophila* differs from that in yeast. Further resolution of the structural of Gpp in complex with nucleosomes will be helpful to address the above questions. Besides, ubiquitination of H2BK123 have been demonstrated to stimulate H3K79 methylation [62]. The above conclusions imply the existence of complex cross-talks between these different histone sites (electronic supplementary material, figure S5C).

In summary, our study shows how the basic patch of the nucleosome exerts its biological functions via a complex network through regulation of histone crosstalk in metazoans. The methods employed and insights provided in this study can be used to explore other regions of the nucleosome (e.g. the acidic patch). Further studies will help to fully understand how the basic patch of histone H4 regulates chromatin dynamics in different tissues and affects transcription of tissue-specific genes.

## Data Availability

Supplementary figures are available as electronic supplementary material. All data supporting the findings of the study are available in the submission. RNA-seq data are available under the accession number PRJNA814824. The data are provided in electronic supplementary material [63].
